# Targeting microbial biofilms using Ficin, a nonspecific plant protease

**DOI:** 10.1038/srep46068

**Published:** 2017-04-07

**Authors:** Diana R. Baidamshina, Elena Y. Trizna, Marina G. Holyavka, Mikhail I. Bogachev, Valeriy G. Artyukhov, Farida S. Akhatova, Elvira V. Rozhina, Rawil F. Fakhrullin, Airat R. Kayumov

**Affiliations:** 1 Kazan Federal University, Institute of Fundamental Medicine and Biology, Kazan, Republic of Tatarstan, Russian Federation; 2 Voronezh State University, Medicine and Biology Faculty, Voronezh, Russian Federation; 3 St Petersburg Electrotechnical University, Biomedical Engineering Research Centre, St. Petersburg, Russian Federation

## Abstract

Biofilms, the communities of surface-attached bacteria embedded into extracellular matrix, are ubiquitous microbial consortia securing the effective resistance of constituent cells to environmental impacts and host immune responses. Biofilm-embedded bacteria are generally inaccessible for antimicrobials, therefore the disruption of biofilm matrix is the potent approach to eradicate microbial biofilms. We demonstrate here the destruction of *Staphylococcus aureus* and *Staphylococcus epidermidis* biofilms with Ficin, a nonspecific plant protease. The biofilm thickness decreased two-fold after 24 hours treatment with Ficin at 10 μg/ml and six-fold at 1000 μg/ml concentration. We confirmed the successful destruction of biofilm structures and the significant decrease of non-specific bacterial adhesion to the surfaces after Ficin treatment using confocal laser scanning and atomic force microscopy. Importantly, Ficin treatment enhanced the effects of antibiotics on biofilms-embedded cells via disruption of biofilm matrices. Pre-treatment with Ficin (1000 μg/ml) considerably reduced the concentrations of ciprofloxacin and bezalkonium chloride required to suppress the viable Staphylococci by 3 orders of magnitude. We also demonstrated that Ficin is not cytotoxic towards human breast adenocarcinoma cells (MCF7) and dog adipose derived stem cells. Overall, Ficin is a potent tool for staphylococcal biofilm treatment and fabrication of novel antimicrobial therapeutics for medical and veterinary applications.

Biofilms are formed by the surface-attached bacterial cells arranged into complex communal tertiary structures and embedded into an extracellular matrix^1^,^2^. The bulk of the matrix is formed by extracellular polymeric substances (EPS) that typically constitute up to 95% of the biofilm and consist of biopolymers (i.e polysaccharides, proteins, lipids and nucleic acids) produced and secreted by the constituent bacteria. The matrix supports the three-dimensional structure of the biofilm and protects the cells from various environmental impacts.

Bacterial cells in biofilms are extremely resistant to medicinal treatment and immune system attacks, that leads to chronic reinfections^1^,^3^,^4^. Many opportunistic bacteria (i.e. *Staphylococcus, Micrococcus, Klebsiella, Pseudomonas*, etc.) form biofilms on chronic and acute dermal wounds impeding their healing, causing reinfection and sepsis^1^,^3^,^4^. Accordingly, the colonization with *S. epidermidis* and/or *S. aureus* is a common cause of intra- and extravascular catheter-associated infection, implants, wound surfaces and mucous membranes^5^. As a result, bacterial biofilms appear a significant clinical challenge leading to increased patient morbidity and mortality from infectious diseases^6^,^7^. Therefore, the prevention of biofilm formation and disruption of already established biofilms is crucially important for clinical treatment of infectious diseases^8^,^9^,^10^.

Destroying the biofilm matrix backbone, for example via enzymatic lysis, is an advantageous approach for biofilms eradication^6^. Numerous bacterial enzymes, such as glycosidases, proteases, and DNases degrade various components of biofilms stimulating cells detachment and increasing cellular susceptibility to antimicrobials^11^. In particular, the glycoside hydrolase dispersin B produced by *Aggregatibacter actinomycetemcomitans* has been shown to sensitize *S. epidermidis* biofilm-embedded cells to antimicrobials action^12^,^13^. Dispersin B injection in combination with triclosan reduced the catheter colonization density by *S. aureus* in rabbits *in vivo*^14^. Another glycoside hydrolase, alginate lyase, successfully enhanced the activity of aminoglycosides against *P. aeruginosa* biofilms both *in vitro*^15^,^16^ and *in vivo*^17^. DNase (NucB) from *Bacillus licheniformis* induced rapid dispersal of biofilm formed by *B. subtilis, E. coli* and *M. luteus*^18^. Recombinant human DNase I (rhDNase) has been shown to disperse preformed *S. aureus* biofilms and increase the susceptibility of *S. aureus* biofilm cells to antiseptics^6^. In addition, two glycoside hydrolases from *Pseudomonas aeruginosa* efficiently destroyed the *Pseudomonas* biofilm backbone^19^.

Proteases are believed to be one of the most effective enzymes in biofilm eradication via hydrolysis of both matrix proteins and adhesins (proteins providing cells attachment onto solid surfaces and other bacteria)^20^,^21^ as well as by the cleavage of signaling peptides of intercellular communication of gram-positive bacteria^22^. Recently, several groups reported the efficacy of proteases as wound healing agents simultaneously exhibiting anti-biofilm properties, such as degradation of the biofilm matrix structural components and destruction of its backbone^23^,^24^,^25^,^26^. The serine protease Esp from *S. epidermidis* has been demonstrated to inhibit the biofilm formation by *S. aureus* and to eradicate the already preformed biofilms^10^. Similar effects have been shown for the elastase LasB from *P. aeruginosa* and proteinase K^10^. Finally, the metalloprotease serratopeptidase (SPEP) produced by *Serratia marcescens* is widely used as an anti-inflammatory agent, successfully inhibiting biofilm formation and enhancing the efficacy of ofloxacin against biofilms of both *P. aeruginosa* and *S. epidermidis*^27^. Two other enzymes, glycosidase pectinase and protease subtilisin A have been shown to suppress the biofilm formation of *Escherichia coli* and enhance the cell sensitivity to ampicillin^24^. Chymotrypsin derived from maggot excretions/secretions was shown to disrupt a protein component of staphylococcal biofilms^28^. The treatment of *Listeria monocytogenes* with sublethal concentrations of serratiopeptidase from *Serratia marcescens* reduced their ability to form biofilms and to invade host cells^29^.

In this paper we show that Ficin (EC 3.4.22.3), a nonspecific sulfhydryl protease isolated from the latex of the *Ficus* tree, disrupts the staphylococcal biofilm backbone, thus significantly increasing the efficiency of conventional antibiotics.

## Results and Discussion

### Staphylococcal biofilms disruption by Ficin

**Figure 1:**
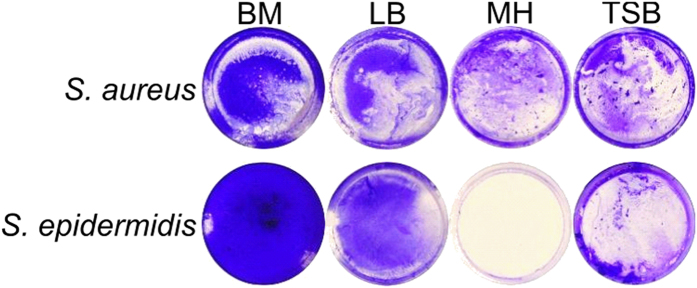
The biofilm formation by *S. aureus* and *S. epidermidis* cultivated in Basal medium (BM), Luria-Bertani broth (LB), Müller-Hinton broth (MH), or Trypticase soy broth (TSB) on 35-mm polystyrol adhesive plates. 72 hours-old biofilms were stained by crystal violet.

**Figure 2:**
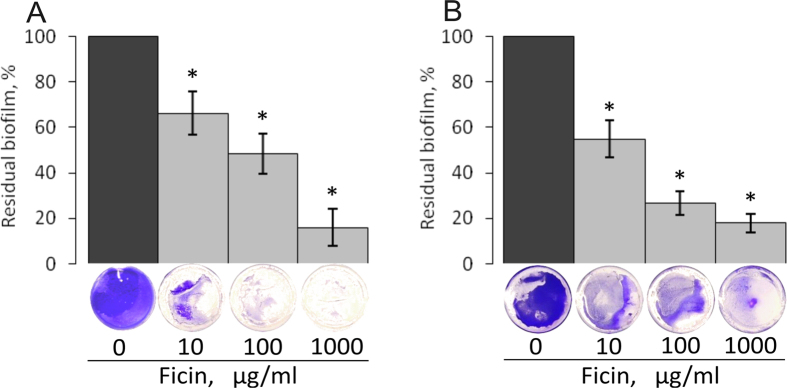
The biofilm disruption by Ficin. *S. aureus* (**A**) and *S. epidermidis* (**B**) were grown in BM broth for 72 h to form a rigid biofilm, the mature biofilms were gently washed by BM and a fresh BM broth was loaded. Ficin was added until final concentrations of 10, 100 or 1000 μg/ml and incubation was followed for 24 h. The residual biofilms were quantified by crystal-violet staining.

Over decades, a number of proteolytic enzymes have been adopted in clinical practice as wound healing agents destroying the cell debris and necrotic tissues. Recently, several proteases were reported to exhibit anti-biofilm properties and to increase the susceptibility of biofilm-embedded bacterial cells to antibiotics^23^,^24^,^25^,^26^. We investigated whether Ficin is able to disrupt bacterial biofilms formed by *S. aureus* and *S. epidermidis*, the bacteria colonizing wounds and thus retarding wound healing^30^. To do so, the bacteria were grown in BM broth earlier developed^31^,^32^ for 72 h on 24-well TC-treated plates that provided a representative and repeatable formation of the rigid biofilm strongly attached to the surfaces, in contrast to Müller-Hinton broth, Trypticase soy broth or LB-medium (Fig. 1). Next the plates were washed twice by fresh BM followed by incubation during 24 h in the fresh BM broth in the presence of Ficin at concentrations of 10, 100 and 1000 μg/ml, since the recommended concentrations of proteolytic enzymes used for wounds healing (like Trypsin and Chymotrypsin) are 1–2 mg/ml^33^,^34^. Then, the culture liquid was discarded and the residual biofilms were quantified by crystal violet staining. The control wells were subjected to all procedures described above except the enzyme addition after the wash and medium replacement. Wells were stained with crystal violet and their absorbance was taken as 100%. Our data indicate that Ficin effectively destroyed the established 3-days old biofilms formed by both *S.aureus* and *S.epidermidis* which can be typically observed on wounds^35^ and cause nosocomial infections (Fig. 2). Even at 10 μg/ml of Ficin only ca. 55–65% of the initial biofilm mass remained as confirmed by crystal violet staining, and biofilms were almost completely eliminated at higher Ficin concentration (1000 μg/ml) (OD_570_ < 0.1). Remarkably, the other proteolytic enzymes such as trypsin or papain could decrease the staphylococcal biofilm for 20–30% only at 100 μg/ml and on 50–60% at 1000 μg/ml^36^ confirming higher efficiency of Ficin for the treatment of staphylococcal biofilms.

To verify the stability of Ficin in the culture liquid, the proteolytic activity was measured using azocaseine as substrate^37^ in wells after the enzyme addition. During the first 4 hours more that 90% of the initial activity was detectable in the liquid (Fig. S1), and approximately half of activity remained in the cultures after 24 hours incubation suggesting high stability of the enzyme.

### The biofilm structure after Ficin treatment

**Figure 3:**
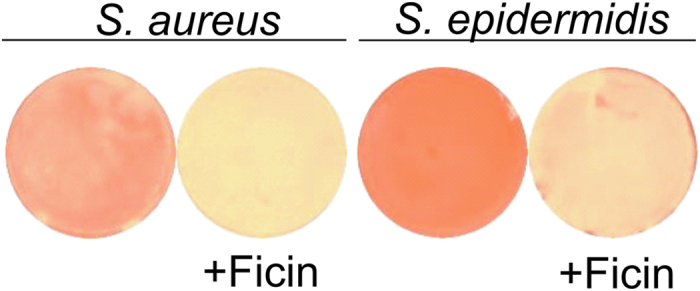
Evaluation of matrix proteins hydrolysis with Ficin. Bacteria were grown on BM medium for 72 h to form a biofilm, then a medium was replaced by the fresh one containing Ficin (1000 μg/ml) and Congo red and incubation was continued for the next 24 h.

To test the hydrolysis of the protein components of the biofilm matrix by Ficin, the preformed 3 day-old biofilms were treated with enzyme in the presence of Congo red, a specific dye staining the amyloid proteins (Fig. 3). The control wells incubated with Congo red in the absence of Ficin were red-stained. In the presence of Ficin a significant decrease of the staining intensity could be observed for both *S. aureus* and *S. epidermidis* plates, indicating the degradation of the protein backbone of the biofilm.

**Figure 4:**
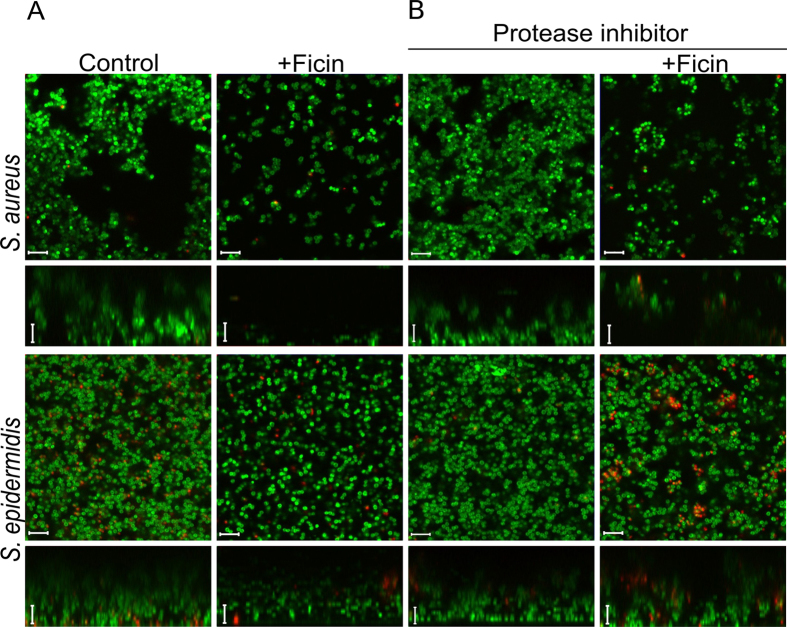
Confocal laser scanning microscopy. *S. aureus* and *S. epidermidis* 48 h-old biofilms were established in cell imaging cover slips (Eppendorf) and treated with Ficin in absence (**A**) or presence (**B**) of protease inhibitors. After 24 h incubation cells were stained with DioC6 and propidium iodide to evaluate the cell viability. The scale bars indicate 5 µm.

Then, to investigate how Ficin affects the biofilm structure, the biofilms of *S. aureus* and *S.epidermidis* treated with Ficin were analyzed by confocal laser scanning microscopy (CLSM). For imaging, both *S. aureus* and *S. epidermidis* were grown for 48 h in 500 μl of BM broth in cell imaging coverglass slides (Eppendorf) to form the biofilm. Then 250 μl of broth was replaced with the fresh aliquote containing Ficin reaching the final concentration of 1000 μg/ml. After 24 h incubation the cells were stained with DioC6 and propidium iodide as described in Materials and Methods and analyzed using CLSM (Fig. 4A).

In control wells the biofilms of both strains reached 20–22 μm and formed pronounced mushroom-shaped structures with cell agglomerates (Fig. 4A, control lane). In the presence of Ficin a significant suppression of the *S. aureus* biofilm was observed, while less pronounced effect was detected for *S. epidermidis.* Also the structure of the biofilm has been changed. Unlike in the control sample, in the Ficin-treated samples a mushroom-like structure of the staphylococcal biofilm disappeared, whereas the uniform layer of the cells could be observed suggesting the destruction of the protein backbone of the matrix. In contrast to the control, this layer could be easily removed by pipetting suggesting its low adherence to the surface. Notably, the fractions of dead cells were rather comparable in wells with or without protease, demonstrating no expressed antimicrobial activity of Ficin and suggesting the absence of a direct evolutionary pressure on the bacterial resistance development.

To verify that the observed changes in the biofilm structure are caused by enzymatic action of Ficin, the established biofilms were also treated with enzyme in the presence of protease inhibitors mix. As shown on Fig. 4B, neither inhibitor alone nor inhibited Ficin caused changes in the biofilm structure and cell viability of *Staphylococci*, confirming that Ficin destroyed the biofilm by hydrolyzing proteins of its matrix.

**Figure 5:**
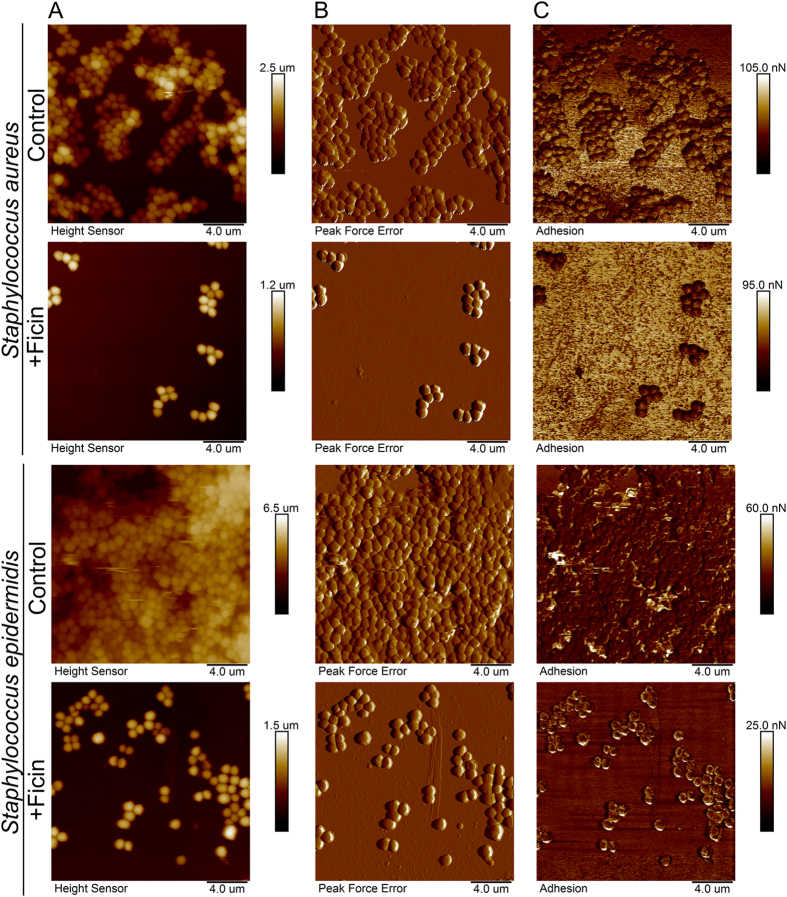
Atomic force microscopy (Peak Force Tapping mode) of intact and Ficin-treated *S. aureus* and *S. epidermidis* biofilms. Bacteria were grown in BM broth for 72 h to form a rigid biofilm, the mature biofilms were gently washed by BM and a fresh BM broth was loaded. Ficin was added until final concentrations of 1000 μg/ml and incubation was followed for 24 h. The residual biofilms were washed, fixed with glutardialdehyde and analyzed with AFM. (**A**) – height (topography); (**B**) – peak force error image; (**C**) –adhesion force image.

For a deeper investigation of the staphylococcal biofilm structural changes after treatment with Ficin, both treated and untreated biofilms were imaged using atomic force microscopy (Fig. 5). AFM data confirms that Ficin treatment leads to efficient eradication of the biofilms. While the overall morphology of the isolated cells in Ficin-treated samples remained unaffected, the cell density was severely reduced. In control samples, the cells formed a typical confluent multilayer biofilm, as shown in AFM images (Fig. 5). Noteworthy, the non-specific tip adhesion mapping showed that the force is almost identical in the cells located in either lower or upper visible biofilm levels. In the case of Ficin-treated biofilms, the AFM imaging revealed the island-like cell clusters on the plate surface. The morphology of Ficin-treated cells was apparently unaffected compared to non-treated cells, while the density of the cell layers was considerably lower. The Peak Force Tapping atomic force microscopy (AFM) allowed to obtain high-resolution images of the quality matching that of the contact mode AFM without damaging the cells. Moreover, unlike in tapping mode AFM, the topography data of microbial cells could be obtained with no typical edge defects due to tip parachuting. Consequently, our AFM topography images of the biofilms represent the precise nanoscale reconstruction of the actual structure of biofilms grown on polymer surfaces confirming the biofilm removal after Ficin treatment.

Further, in *S. aureus* biofilms treated with Ficin the non-specific adhesion of non-functionalized AFM probe tip was somewhat reduced, unlike in intact biofilms, indicating that the specific adhesion of the cells to substrates might be also reduced. On the other side, only 2–4 fold decrease of *S. epidermidis* and *S. aureus* biofilms layer after Ficin treatment was observed in CLSM microphotographs, while both crystal violet and Congo red quantification showed 5–7-fold reduction of the biofilm (Figs 2 and 3). Since the AFM images demonstrated a monolayer of residual cell clusters on the surface, we hypothesized that the 5–8 μm layer observed with CLSM (Fig. 4) might represent the sedimented cells which are not well-adherent to the surface anymore. This hypothesis is partially confirmed by the non-specific tip adhesion AFM data for Ficin-treated samples, which appeared to be somewhat lower when compared to control samples, indicating that the specific adhesion of the cells to the substrates might also be reduced. Non-specific adhesion forces of cell surfaces to silicon nitride AFM tips by no means can be directly extrapolated onto the adhesive properties of bacteria to real substrates. However, this may serve as a good indicator of certain physiological effects occurring in Ficin-treated bacteria forming biofilms. Together with Congo red staining data (Fig. 2) and cell density observations (Fig. 3) from the Peak Force Tapping AFM nanomechanical data (Fig. 5) this suggests that Ficin apparently hydrolyses both the biofilm matrix and proteins participating in the adhesion of microbial cells, thus significantly reducing their ability to form biofilms as shown for other proteases.

### Ficin treatment enhances the efficacy of antimicrobials against biofilm-embedded *Staphylococci*

After being embedded into the matrix of the biofilm, bacteria become almost inaccessible for biocides and antibiotics. We tested whether Ficin would increase the efficiency of antibiotics against surface-adherent bacteria due to the biofilm damage. Both *S. aureus* and *S. epidermidis* strains were sensitive to ciprofloxacin according to EUCAST rules (http://mic.eucast.org/), therefore this antibiotic was chosen as a model antimicrobial drug. The MIC values of ciprofloxacin established by the broth microdilution method were 2 μg/ml for *S.aureus* and 1 μg/ml for *S. epidermidis*. The MBCs were 8 μg/ml and 4 μg/ml, respectively.

**Figure 6:**
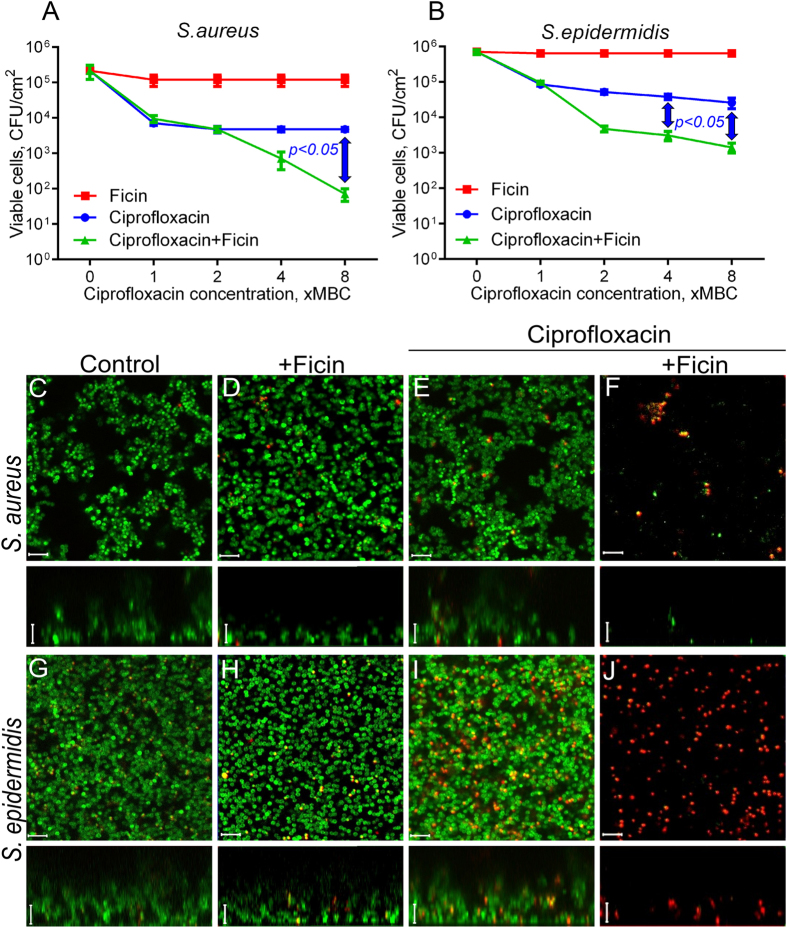
The Ficin treatment increases the efficacy of ciprofloxacin against biofilm-embedded *Staphylococci.* Ficin (1000 μg/ml) and ciprofloxacin (1–8 × MBC) were added to 48 hours-old biofilms of *S. aureus* and *S. epidermidis*. After 24 h incubation, the biofilms were washed twice with sterile 0.9% NaCl. The adherent cells were scratched, resuspended and their viability was analyzed by using drop plate assay (**A**,**B**). Alternatively, 48 hours-old biofilms of *S. aureus* and *S. epidermidis* were incubated 24 h in presence of Ficin (1000 μg/ml) and ciprofloxacin (8 × MBC) in cell imaging coverglass slides and analyzed with confocal scanning microscopy (**C**–**J**). Significant differences between 10 log_10_ of the viable cell counts after treatment with ciprofloxacin in either absence of presence of Ficin according to Pearson’s Chi-squared homogeneity test (*p* < *0.05*) are indicated in the figure. The scale bars indicate 5 µm.

To test the effect of ciprofloxacin on *S. aureus* and *S. epidermidis* biofilm-embedded cells in the presence of Ficin, 48-h biofilms were prepared on 96-well TC-treated plates. The established biofilms were washed twice by fresh BM broth to remove non-adherent cells, and incubated for the next 24 h in the fresh BM broth in the presence of Ficin and ciprofloxacin as indicated (Fig. 6, Fig. S2). Ciprofloxacin was added to the final concentrations of 1×, 2×, 4× and 8× MBCs, the final concentration of Ficin was fixed at 1000 μg/ml. After 24 h incubation, the culture liquids with planktonic and detached cells were saved, the biofilms were washed twice by sterile 0.9% NaCl. Then the viability of both detached and biofilm-embedded cells was analyzed by drop plate assay. The experiments were carried out in biological triplicates with three independently treated samples in each one, the latter being averaged in each biological replicate, the differences between groups were analyzed by using Pearson’s Chi-squared test and were considered significant at p < 0.05.

The viability of both *S. aureus* and *S. epidermidis* cells in either biofilm or culture liquid was insignificantly affected by the enzyme (Fig. 6, Fig. S2). When ciprofloxacin was added into the broth, its 8 × MBC reduced the amount of *S. aureus* and *S. epidermidis* detached cells by nearly 2 and 3 orders of magnitude, respectively (Fig. S2). In the presence of Ficin half of the initial antibiotic concentration was required to achieve the same effect, probably, due to the possible disintegration of detached bacterial clumps by the enzyme. Significant differences between ciprofloxacin-treated cells in presence or absence of Ficin were observed at 8 × MBC of antibiotic. The CFUs of the biofilm-embedded cells of both strains decreased only 10-fold in the presence of ciprofloxacin even at 8 × MBC, while in the presence of Ficin the decrease up to 3 orders of magnitude could be observed (Fig. 6) with significance of 0.05 at 8 × MBC for *S. aureus* and 4–8 × MBC for *S. epidermidis*. At lower Ficin concentration (100 μg/ml) the increase of ciprofloxacin efficacy was also observed although less pronounced (not shown). The increase of ciprofloxacin efficacy against biofilm-embedded *Staphylococci* was also verified using the confocal laser scanning microscopy. For that, the cells were grown for 48 h in 500 μl of BM broth in cell imaging coverglass slides (Eppendorf) to prepare the biofilm. Then 250 μl of broth was replaced with the fresh one containing Ficin (1000 μg/ml) and ciprofloxacin (8 × MBC). After 24 h cultivation the cells were stained with DioC6 and propidium iodide, as described in Materials and Methods, and analyzed with CLSM (Fig. 6). In wells containing only ciprofloxacin most cells were stained in green suggesting their viability (Fig. 6D,H), with only a few dead cells being detectable. In contrast, in the wells with both antibiotic and protease nearly no viable cells could be detected, and considerably reduced quantity of red-stained cells could be observed.

**Figure 7:**
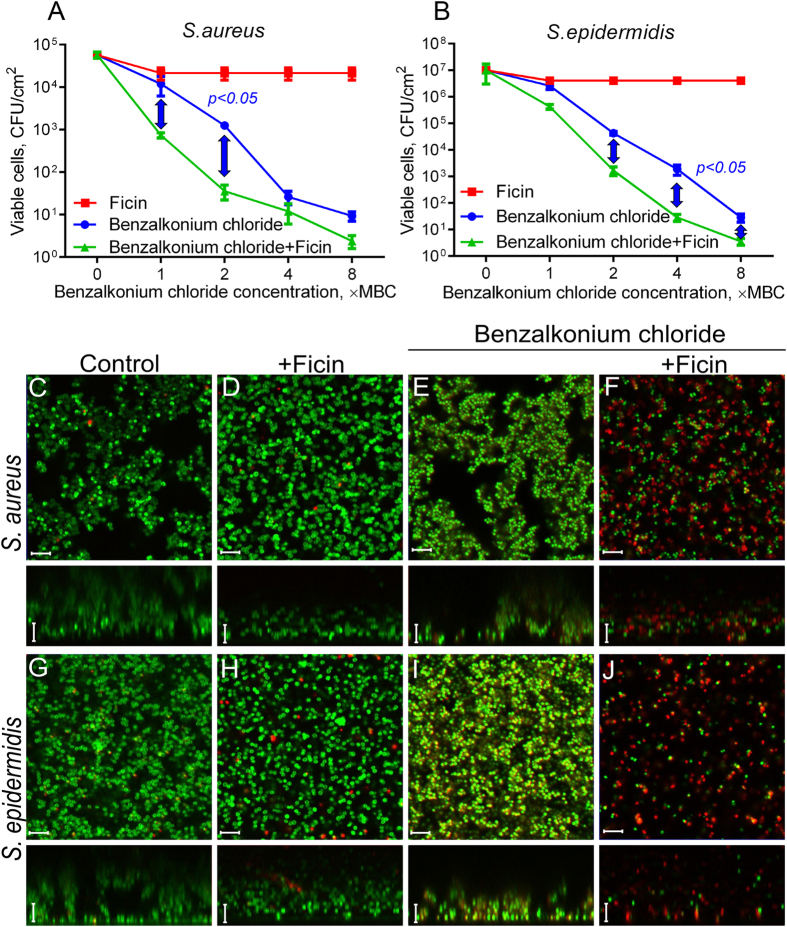
The Ficin treatment increases the efficacy of benzalkonium chloride against biofilm-embedded *Staphylococci.* Ficin (1000 μg/ml) and benzalkonium chloride (1–8 × MBC) were added to 48 hours-old biofilms of *S. aureus* and *S. epidermidis*. After 24 h incubation, the biofilms were washed twice with sterile 0.9% NaCl. The adherent cells were scratched, resuspended and their viability was analyzed by using drop plate assay (**A**,**B**). Alternatively, 48 hours-old biofilms of *S. aureus* and *S. epidermidis* were incubated 24 h in presence of Ficin (1000 μg/ml) and benzalkonium chloride (8 × MBC) in cell imaging coverglass slides and analyzed with confocal scanning microscopy (**C**–**J**). Significant differences between 10 log_10_ of the viable cell counts after treatment with benzalkonium chloride in either absence of presence of Ficin according to Pearson’s Chi-squared homogeneity test (*p* < *0.05*) are indicated in the figure. The scale bars indicate 5 µm.

The efficiency of other antimicrobials regularly used for outer treatment of wounds also increased in presence of Ficin. In particular, Ficin treatment led to the twofold decrease of the efficient concentration of Benzalkonium chloride, the biocide belonging to quaternary ammonium salts (Fig. 7, Fig. S3). Here, the significant differences between Ficin treated and untreated cells were observed at low concentrations of antimicrobial (1–2 × MBC) for both detached and biofilm-embedded cells. Similar effect could be observed for gentamycin (Fig. S4), although less pronounced, probably due to the low sensitivity of strains used to this antimicrobial.

**Figure 8:**
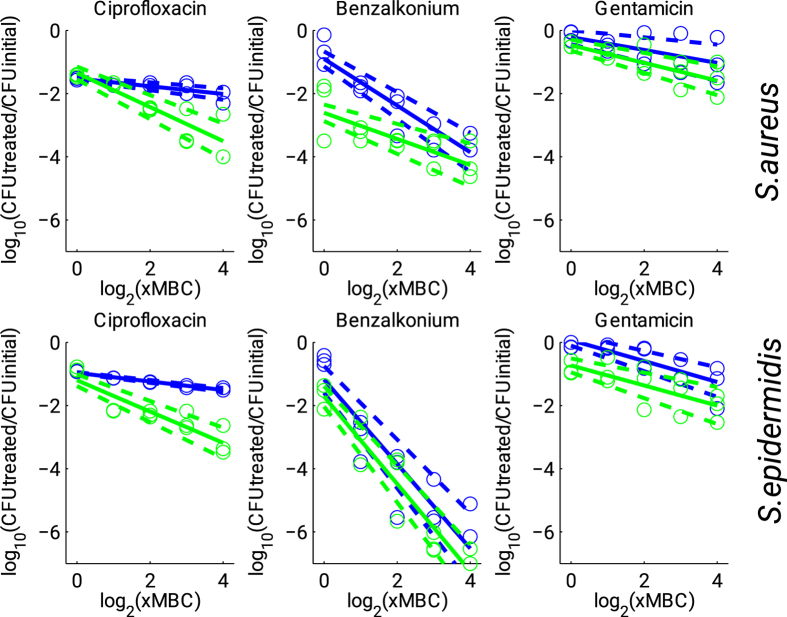
Dose-response curves for biofilm-embedded *Staphylococci* treated with antimicrobials in either presence (green) or absence (blue) of Ficin (1000 μg/ml). Full lines denote regression lines, while dashed lines denote corresponding 95% confidence intervals.

To analyze the antimicrobial enhancement efficiency in the presence of Ficin in more details, respective dose-response curves were plotted providing residual CFUs function as a function of antibiotic concentrations (see Fig. 8, upper panel for *S. aureus*, lower panel for *S. epidermidis*). Rough estimates of the dose-response curves were obtained by linear regression applied in logarithmic scale. Figure 8 shows that the addition of Ficin significantly increased the sensitivity of both *S. aureus* and *S. epidermidis* cells to Ciprofloxacin leading to 10-fold discrepancy for 8 × MBC. In contrast, for Benzalkonium chloride and Gentamicin treatment of both *S. aureus* and *S. epidermidis* cells, the effect of Ficin was clearly observed already at 1 × MBC, likely indicating higher susceptibility of the biofilm-embedded cells to respective antibiotics. To achieve comparable effect without Ficin treatment, the antibiotic concentrations had to be increased 4- to 16-fold. Accordingly, our results indicate that treatment with Ficin reduces the required antimicrobial dose likely due to the increased susceptibility of the biofilm-embedded cells.

For detached cells (see Fig. S5 in the Supplementary Information available), the above effects were less pronounced, and the discrepancy between cells treated with either antibiotics and ficin or with antibiotics alone was less significant, while still some limited enhancement of treatment efficacy could be observed at large antibiotic concentrations, probably due to the destruction of detached cell clumps by Ficin.

Altogether, these observations suggest that Ficin destroys the biofilm backbone making the cell accessible for antimicrobials. Similar effect has been observed previously for subtilisin A and some 2(5 *H*)-furanone derivatives^38^,^39^, suggesting that disruption of biofilms could be one of the factors of how proteases speed the wound healing. Furthermore, a significant decrease in the bacterial biofilm thickness was observed, this way confirming that the biofilm was nearly completely eradicated and suggesting the combination of the Ficin with antibiotics as a promising approach for the development of wounds treatment therapeutics.

### Cytotoxicity evaluation

To investigate the cytotoxicity of Ficin, the metabolic MTS-assay was performed employing MCF7 cells, human skin fibroblasts and dog adipose derived stem cells (ADSC) (see Table 1). No suppression of the dehydrogenase activity by the enzyme was detected within the concentrations tested after the cells were treated by the enzyme for 24 h. Additionally, to test the influence of long-term Ficin treatment, the carcinoma and stem cells were grown in the presence of Ficin samples over 3 days. After every 24 h the culture liquid was removed from part of the wells and cells were live/dead stained and analyzed with differential fluorescence microscopy using Carl Zeiss Observer 2.0 microscope. No significant increase in the fraction of necrotic MCF7 or stem cells (see Figs S6 and S7) was detected in either control wells or wells with Ficin at concentrations of 10–1000 μg/ml, indicating Ficin safety for potential biomedical applications at least under conditions been tested.

## Conclusion

Our results confirm that Ficin, a nonspecific sulfhydryl protease from *Ficus* tree, effectively disrupts the biofilm matrix backbone of *S. aureus* and *S. epidermidis,* which colonize skin, catheters and cause nosocomial infections. The efficiency of biofilm disruption activity has been also confirmed using atomic force and fluorescence microscopy of treated and non-treated biofilms. As a result, the presence of protease led to at least twofold decrease of antimicrobials (ciprofloxacin and benzalkonium chloride) concentrations required to reduce the number of viable biofilm-embedded cells. Ficin is not cytotoxic, as we have confirmed using viability assays with adipose derived stem cells and MCF7 carcinoma cells. Importantly, Ficin did not affect the growth rate and morphology of either cell lines. We believe that Ficin appears a safe and effective agent for external wound treatment to suppress the biofilm formation and reduce the reinfection risk. Although the detailed investigation of the practical aspects of wound healing with Ficin requires further thorough investigations, our current results indicate that Ficin is an advantageous tool for therapeutic antibiofilm treatment.

## Materials and Methods

A commercially available Ficin obtained from MP Biomedicals, USA (0.2 U/mg) was used in this study.

### Bacterial strains and growth conditions

*Staphylococcus aureus subsp. aureus* (ATCC^®^ 29213™) and *Staphylococcus epidermidis* (clinical isolate, obtained from the Kazan Institute of Epidemiology and Microbiology, Kazan, Russia) were used for the biofilm assays. Bacterial strains were cultivated using LB medium. The Müller-Hinton broth (Fluka) or Trypticase soy broth (Sigma) did not provide stable biofilm formation by both *Staphylococcus aureus* and *Staphylococcus epidermidis*, as is has been determined in preliminary studies (Fig. 1), thus the modified Basal medium (BM) (glucose 5 g, peptone 7 g, MgSO_4_ × 7H_2_O 2.0 g and CaCl_2_ × 2H_2_O 0.05 g in 1.0 liter tap water) was used for the biofilm formation assays^31^,^39^. Bacteria were grown for 48–72 hours as indicated under static conditions at 37 °C to obtain rigid biofilm structures.

### Biofilm staining

To investigate the effect of Ficin on bacterial biofilms, a bacterial suspension (2–9 × 10^6^ CFU ml^−1^) was inoculated in BM broth and grown in 96-well plates (200 μl per well) or 34-mm plates (2 ml per plate). All plates (polystyrol) were TC-treated and obtained from Eppendorf. After 72 h of growth the biofilm was formed, the old medium was exchanged by the new one, the Ficin was added and the incubation was continued for the next 24 h. To analyze the hydrolysis of the protein backbone of the biofilm matrix by Ficin, a Congo Red solution^40^ (final concentration 50 μg/ml) was added to the preformed biofilm together with Ficin.

For crystal violet staining, the culture supernatant was discarded, and the wells were washed several times with phosphate-buffered saline (PBS) to remove non-adherent cells. The samples obtained were then stained with crystal violet as described previously^41^. Briefly, the plates were air dried for 20 min, and the surface-attached cells were stained with 200 μl of 1% crystal violet solution for 20 min. Subsequently, the crystal violet was removed and the plates were washed 3 times with tap water. After 30 min of air drying, 200 μl or 2 ml of 96% ethanol was added to dissolve the cell-bound crystal violet, and the absorbance was measured at 570 nm using the microplate reader Tecan Infinite 200 Pro. The wells incubated with the cell-free medium were also stained and used as a reference.

### Evaluation of antibacterial activity

The minimum inhibitory concentration (MIC) of antimicrobials was determined by the broth microdilution method in BM broth in 96-well non-treated cell culture plates (Eppendorf) in three independent repeats. The concentrations of antibiotic after a series of two-fold dilutions were in the range of 0.5–512 μg/ml. Wells were seeded with 200 μl of the bacterial culture (3 × 10^7^ CFU/ml) and incubated at 37 °C. The MIC was determined as the lowest concentration of compound for which no visible bacterial growth could be observed after 24 h of incubation. To determine the minimum bactericidal concentration (MBC), 5 μl of culture liquid from the wells with no visible growth were inoculated into 5 ml of LB broth and cultivated for 24 h. The MBC was determined as the lowest concentration of compound for which no visible bacterial growth could be observed.

To evaluate the antibacterial activity against biofilm-embedded cells, rigid biofilms were preformed by 48 h growth in BM broth as indicated, the plates were washed twice with sterile broth, followed by the exchange of the old medium by the new one. Ficin and antibiotics were added as indicated and the incubation was continued for the next 24 h. The viability of both biofilm-embedded and biofilm-detached cells in culture liquid was investigated by both drop plate approach and CLSM.

### Drop plate assay

To evaluate the viability of detached and planktonic cells with drop plate assay, a series of 10-fold dilutions of liquid culture from each well were prepared in 3 technical repeats and 50 μl of suspension was dropped by 10 μl-drops onto LB plates^42^. CFUs were counted from the two last drops containing countable amount of colonies and averaged. To evaluate the viability of biofilm-embedded cells, the wells were washed twice with 0.9% NaCl to remove the non-adherent cells, and the biofilms were suspended in 0.9% NaCl by scratching the well bottoms with subsequent treatment in an ultrasonic bath for 2 min to facilitate the disintegration of bacterial clumps. Viable cells were counted by the drop plate method as described above.

### Biofilm assay with CLSM

To evaluate the viability of biofilm-embedded cells, bacterial suspension was inoculated in BM broth and grown on cell imaging cover slips (Eppendorf) under static conditions. After 48 h of growth, half of the medium was exchanged by the fresh medium. Next Ficin and antimicrobials were added as described previously and further incubated for 24 h. The samples were then stained for 5 min with the 3,3′-Dihexyloxacarbocyanine iodide (Sigma) at final concentration of 0.02 μg/ml (green fluorescence) and propidium iodide (Sigma) at final concentration of 3 μg/ml (red fluorescence) to differentiate between bacteria with intact and damaged cell membranes (live and dead cells). Confocal laser scanning microscopy images (CLSM) were obtained with a Carl Zeiss LSM 780 confocal microscope with Ζ-series images taken in 1-μm slices.

### Atomic force microscopy (AFM)

Atomic force microscopy images of the air-dried microbial biofilms were collected using Dimension Icon scanning probe microscope (Bruker, USA) operating in PeakForce Tapping™ mode. For AFM imaging in air the biofilms were grown in BM-broth on 34-mm plates (TC-treated, Eppendorf, 2 ml per plate) and treated with Ficin as described above. Then the treated biofilms were washed with water and fixed with glutaraldehyde (0.1% aqueous solution) for 4 hours. After subsequent washing with water the plates were dried in air and imaged at ambient conditions. Scan Asyst-Air probes (Bruker) having nominal length 115 μm, tip radius 2 nm, spring constant 0.4 N\m were used throughout. The images were obtained at 512 lines\scan at 0.8–0.9 Hz scan rate. The images were acquired in height (topography), peak force error and adhesion channels. The raw AFM imaging data obtained were processed and analysed using Nanoscope Analysis v.1.7 software (Bruker).

### Cytotoxicity assay

The MCF7 cells and dog adipose derived stem cells^36^ were cultured in DMEM supplemented with 10% FBS, 2 mM L-glutamine, 100 μg/ml penicillin and 100 μg/ml streptomycin. The cells were seeded in 96-well plates at the density of 3000 cells per well and allowed to attach overnight. The cells were cultured at 37 °C and 5% CO_2_ in the presence of Ficin. After 48 h of cultivation the cells were subjected to MTS-assay based on the cellular reduction of MTS (3-(4,5-dimethyl-2-yl)-5-(3-carboxymethoxyphenyl)-2-(4-sulfophenyl)-2H-tetrazolium) by the mitochondrial dehydroxygenase using phenazine methosulfate (PMS) as the electron coupling reagent (Promega Cell Proliferation Assay kit). The MTS tetrazolium compound was bioreduced by the viable cells into a colored formazan product which was measured using Tecan Infinite 200Pro at 550 nm.

### Statistical analysis

Experiments were carried out in biological triplicates (i.e. newly prepared cultures and medium) with 3 independent repeats in each one. The fraction of not viable cells was estimated as the relative fraction of the red cells among all cells in the combined images obtained by overlaying of the green and the red fluorescence microphotographs (10 images per each sample). The statistical significance of the biofilm destruction in the series of Ficin-treated samples was assessed using the Mann-Whitney U-test for independent samples separately for each of three tested enzyme concentrations. Since the drop plate assay results were assessed from 10-fold dilutions, where typically only in the two latter dilutions the number of colonies was countable, to assess the statistical significance, we compared 10 log_10_(*c*), where *c* is the obtained cell number, using the Pearson’s chi-squared homogeneity test. For both tests significant differences were reported at *p* < 0.05. Dose-response curves were estimated by linear regression in the logarithmic scale for both × MBC and relative CFU counts, with 95% confidence intervals for the regression coefficients.

## Additional Information

**How to cite this article**: Baidamshina, D. R. *et al*. Targeting microbial biofilms using ficin, a nonspecific plant protease. *Sci. Rep.* **7**, 46068; doi: 10.1038/srep46068 (2017).

**Publisher's note:** Springer Nature remains neutral with regard to jurisdictional claims in published maps and institutional affiliations.

## Supplementary Material

Supplementary Information

## Figures and Tables

**Table 1:** Cytotoxicity of Ficin in metabolic MTS test (residual activity, percentage of the solvent control).

Final concentration of Ficin, μg/ml	MCF7 cells			Dog adipose derived stem cells		
	10	100	1000	10	100	1000
Residual activity of dehydrogenase, %	122 ± 12.3	83 ± 12.5	105 ± 7.9	98 ± 0.21	90 ± 0.21	83 ± 0.21
