# Supplementary material for: Targeting microbial biofilms using Ficin, a nonspecific plant protease
Source: Sci Rep. 2017 Apr 7;7:46068. doi: 10.1038/srep46068 (PMC5384253; doi:10.1038/srep46068)
Supplement: Supplementary Information [file srep46068-s1.pdf]

Supplementary file

Targeting microbial biofilms using ficin, a nonspecific plant protease

Diana R. Baidamshina[1,+], Elena Y. Trizna[1,+], Marina G. Holyavka[2], Mikhail I. Bogachev[3], Valeriy G. Artyukhov[2], Farida S. Akhatova[1], Elvira V. Rozhina [1], Rawil F. Fakhrullin[1] and Airat R. Kayumov[1, *]

[1]Kazan Federal University, Kazan, Republic of Tatarstan, Russian Federation
[2]Voronezh State University, Voronezh, Russian Federation
[3]St Petersburg Electrotechnical University, St. Petersburg, Russian Federation
* kairatr@yandex.ru, 8(843)2337802
[+]these authors contributed equally to this study

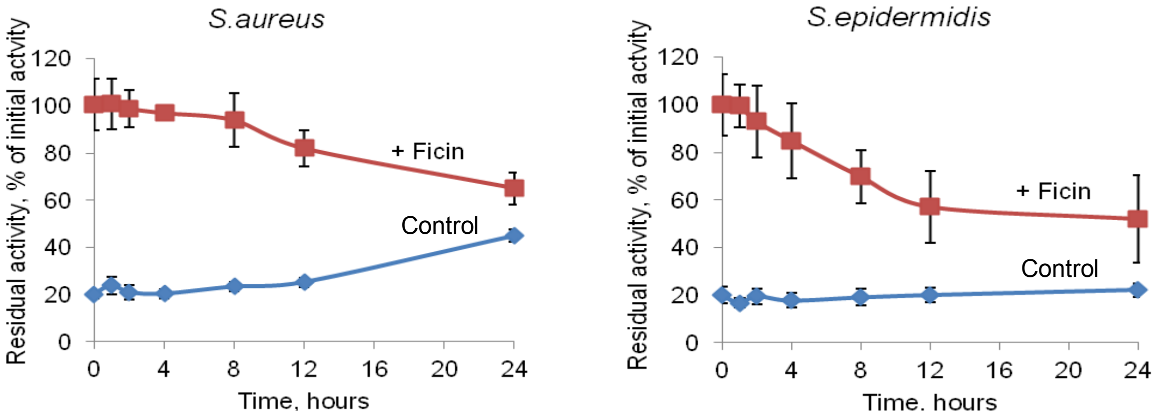

Figure S1. The Ficin stability in culture liquids of *Staphylococci.*

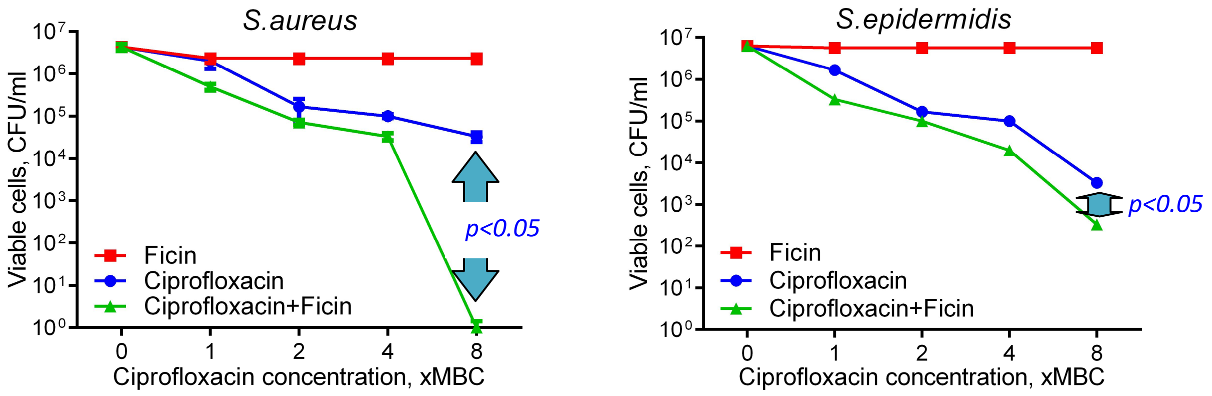

Figure S2. The Ficin treatment increases the efficacy of ciprofloxacin against biofilm-detached *Staphylococci.* Ficin (1000 µg/ml) and ciprofloxacin (1-8×MBC) were added to 48 hours-old biofilms of *S. aureus* and *S. epidermidis*. After 24 h incubation, the cells viability in the culture liquids was analyzed by using drop plate assay. Significant differences between 10 $\log_{10}$ of the viable cell counts after treatment with ciprofloxacin in either absence of presence of Ficin according to Pearson's Chi-squared homogeneity test ($p<0.05$) are indicated in the figure.

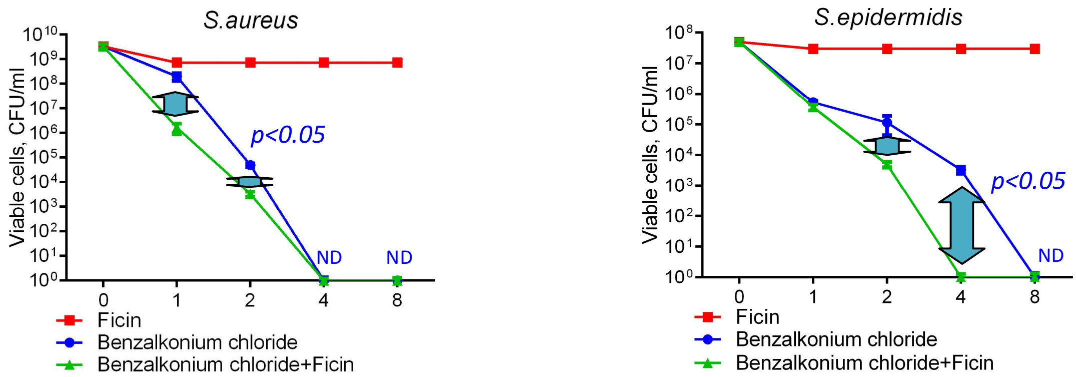

Figure S3. The Ficin treatment increases the efficacy of benzalkonium chloride against biofilm-detached *Staphylococci.* Ficin (1000 µg/ml) and benzalkonium chloride (1-8×MBC) were added to 48 hours old biofilms of *S. aureus* and *S. epidermidis*. After 24 h incubation, the cells viability in the culture liquids was analyzed by using drop plate assay. Significant differences between 10 $\log_{10}$ of the viable cell counts after treatment with benzalkonium chloride in either absence of presence of Ficin according to Pearson's Chi-squared homogeneity test ($p<0.05$) are indicated in the figure. ND-not determined.

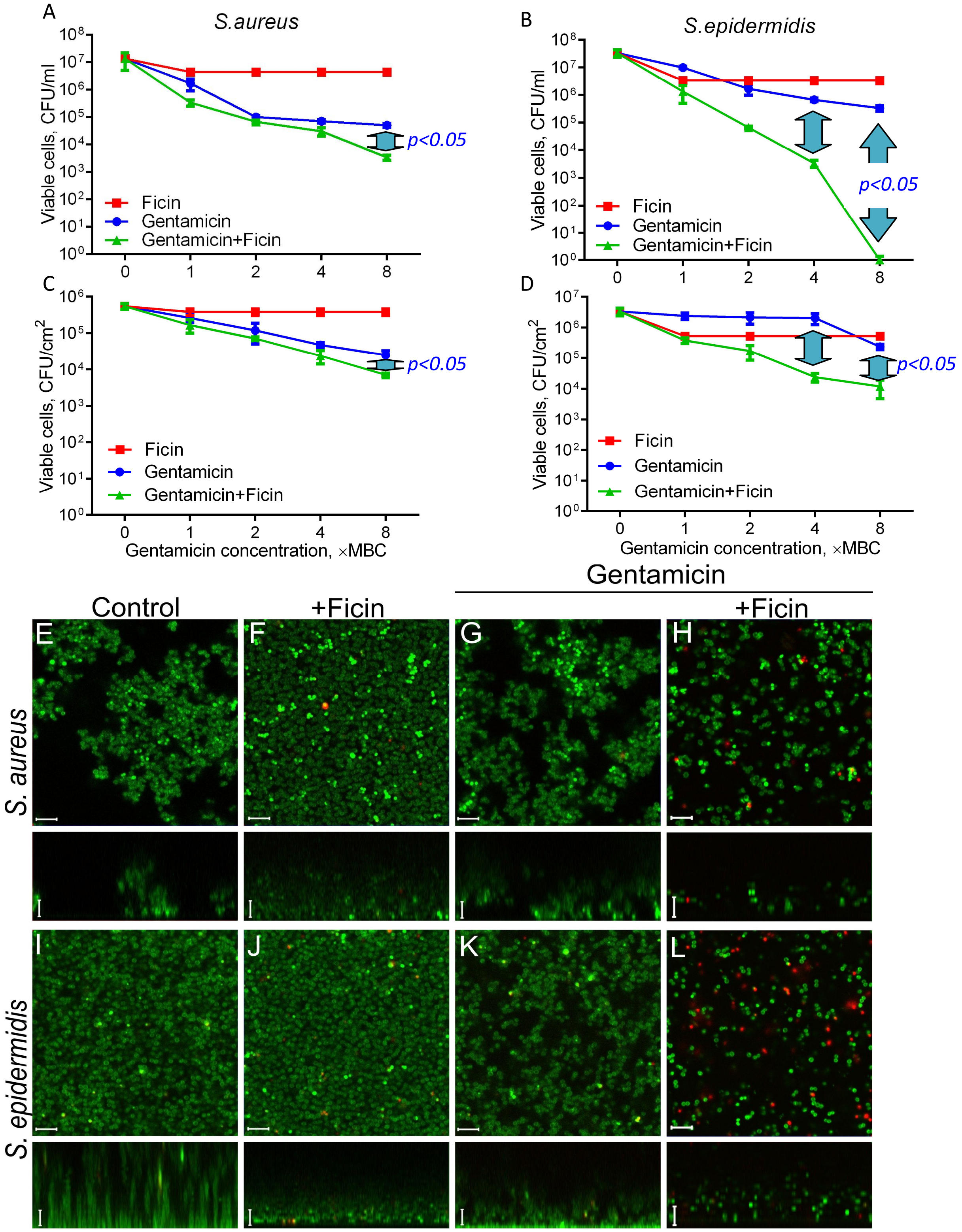

Figure S4. The Ficin treatment increases the efficacy of gentamicin against *Staphylococci.* Ficin (1000 µg/ml) and gentamicin (1-8×MBC) were added to 48 hours-old biofilms of *S. aureus* and *S. epidermidis*. After 24 h incubation, the biofilms were washed twice with sterile 0.9% NaCl. The cells viability in the culture liquids was analyzed by drop plate assay (A, B). The adherent cells were scratched, resuspended and their viability was also analyzed by using drop plate assay (C, D). Alternatively, 48 hours-old biofilms of *S. aureus* and *S. epidermidis* were incubated 24 h in presence of Ficin (1000 µg/ml) and gentamicin (8×MBC) in cell imaging coverglass slides and analyzed with confocal scanning microscopy (E-L). Significant differences between 10 $\log_{10}$ of the viable cell counts after treatment with gentamicin in either absence of presence of Ficin according to Pearson's Chi-squared homogeneity test ($p<0.05$) are indicated in the figure.

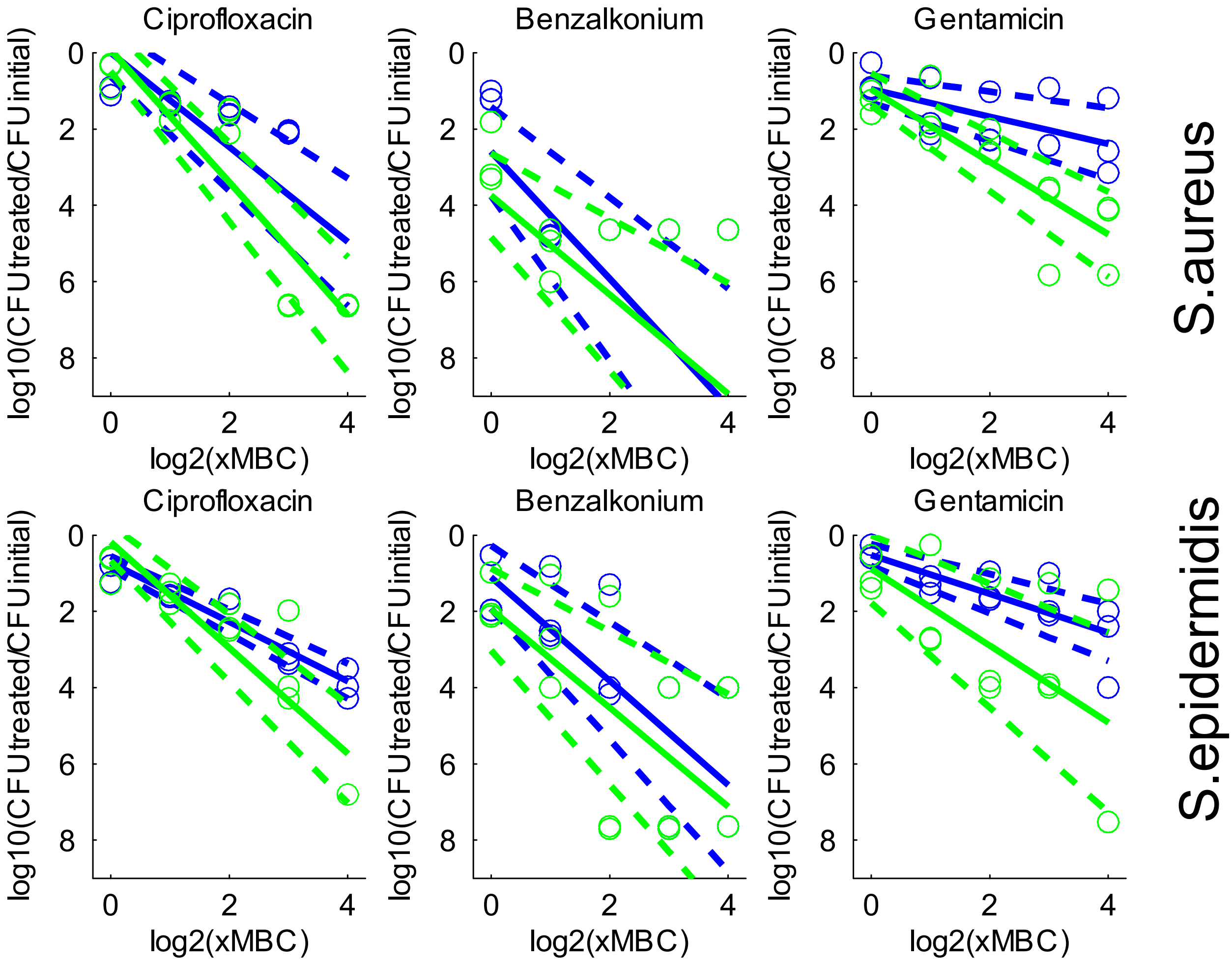

Figure S5. Dose-response curves for biofilm-detached *Staphylococci* treated with antimicrobials in either presence (green) or absence (blue) of Ficin (1000 µg/ml). Full lines denote regression lines, while dashed lines denote corresponding 95% confidence intervals.

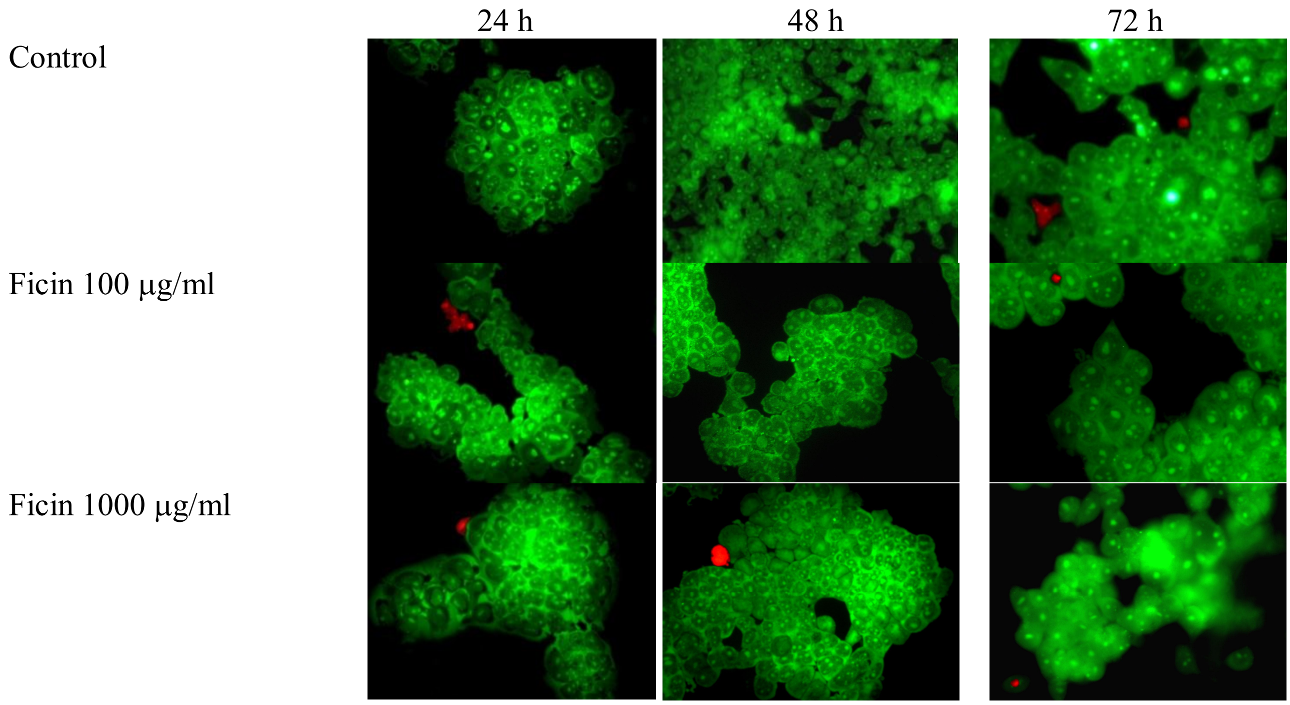

Figure S6. The proliferation of MCF7 cells in the presence of Ficin (magnification 400×).

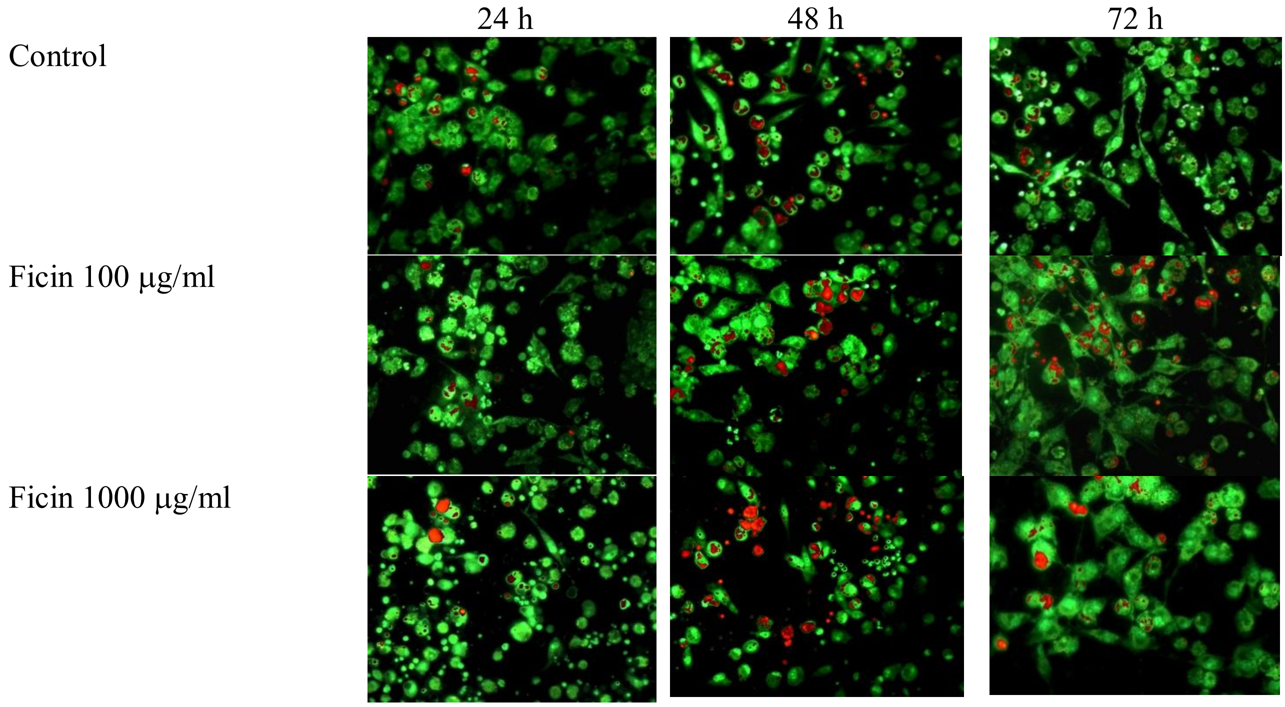

Figure S7. The proliferation of dog adipose derived stem cells (ADSC) in the presence of Ficin (magnification 400×).
